# Light People: Professor Guangming Tao

**DOI:** 10.1038/s41377-022-00995-2

**Published:** 2022-10-19

**Authors:** Hui Wang

**Affiliations:** Changchun Institute of Optics, Fine Mechanics and Physics, Chinese Academy of Sciences, 3888 Dong Nan Hu Road, Changchun, 130033 China

**Keywords:** Metamaterials, Optical techniques

## Abstract

‘In the short but hot summer nights, only by opening doors and windows can the room be cooled down slightly’ complained the famous Chinese poet Fu Du in his poem *Summer Night Lament* more than 1000 years ago. Currently, people are suffering from the summer heat, with ambient temperatures around or exceeding 40 °C. Phrases such as “killer hot days” are no longer exaggerated statements. Hot weather can not only frustrate people, but also make people sick or even endanger lives owing to heatstroke. Fortunately, scientists have come up with a great invention, which will provide us with some much-needed relief in hot summers—the optical metafabric. The magic of this innovation is that, although it feels just like a normal fabric and has all the necessary properties of a wearable fabric, a garment made with it can cool down one’s body by nearly 5 °C even under direct sunlight, making it ideal for summer clothing. In the last few years, a team led by Prof. Guangming Tao of Huazhong University of Science and Technology has achieved remarkable progress in the area of optical metafabrics, finding new applications for this material and making it more affordable. Now, please follow our *Light* Science Editor as he explores this amazing new material and finds how it has been used in the industry, society, and Winter Olympics.

**Biography:** Guangming Tao is a Professor at Wuhan National Laboratory of Optoelectronics, and the School of Materials Science and Engineering, the Director of Sports and Health Initiative, Optics Valley Laboratory, the Chief Scientist in Biomedical Materials and Life Support Equipment Division of Institute of Medical Equipment Science and Engineering, Huazhong University of Science and Technology. He is committed to interdisciplinary research of fiber optoelectronics. He has published 80+ papers in journals including *Science* (1), *Nature* (2), holds 30+ granted and 40+ pending patents (9 licensed/transferred). Professor Tao’s research team received the 2021 Award for China’s Top 10 Optical Breakthroughs, Top 10 Social Impact Events in China’s Optics Field in 2021, and the Candidate Projects of China Issues Top 10 Scientific Advances of 2021, etc. His research on Fiber Optoelectronics has been widely reported by mainstream media, such as People’s Daily, Xinhua News, CCTV-13, CCTV-10, the official station of the Ministry of Science and Technology of the People’s Republic of China, Science Daily, Science News, American Chemical Society, and American Physical Society.

**Figure:**
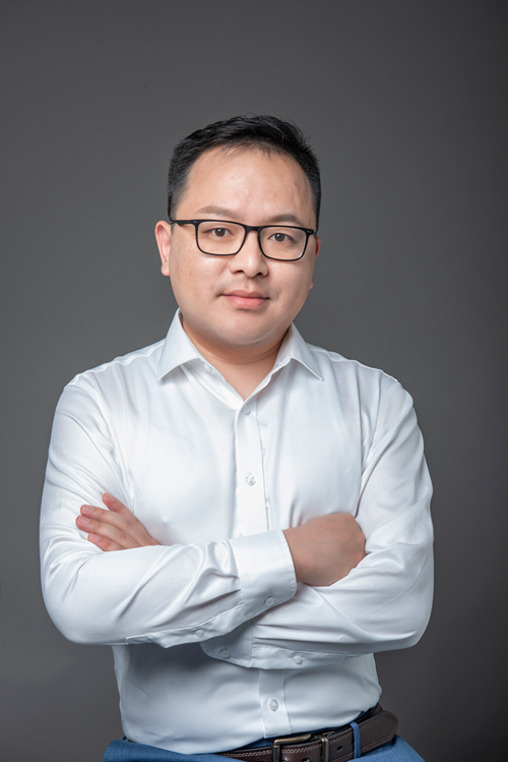

**1. Could you briefly introduce your current research area and latest progress?**

Prof. Tao: My current research focuses on interdisciplinary research on fibre optoelectronics, including passive thermal management fabrics and intelligent fabric spaces.

In passive thermal management fabrics, the passive thermal management is realised *via* the spectral engineering of fabrics. We designed and produced a hierarchical morphology cooling metafabric for high-efficiency outdoor personal thermal management. In addition, to meet the need of a passive warming technology for face protection in cross-country skiing, we developed a passive warm face mask to assist athletes in preparing for the Olympic Winter Games in Beijing 2022.

In an intelligent fabric space, the monitoring of human behaviour and health status requires multi-modality, high-density distributed imperceptible sensors in a certain space. In our imperceptible intelligent bed project, we deployed substantial flexible sensors, combined with edge computing and digital twins, to achieve health monitoring with the integration of sensing, transmission, and computing technologies. Currently, our intelligent bed can monitor fundamental physiological indicators such as the motion mode, heart rate, respiration, and body temperature, providing users with timely health warnings and long-term auxiliary diagnosis. For motion perception, we developed a series of intelligent sport equipment, such as digital boxing gloves and intelligent training assistant sandbags. Based on these motion-sensing devices, we implemented real-time motion information monitoring, such as the athlete’s gait, speed and trajectory of the punch, and punch power. As the co-founder and vice president of the Hubei Province Boxing Association, I think this work is critical to the judging of boxing and status analysis in daily training.

**Figure:**
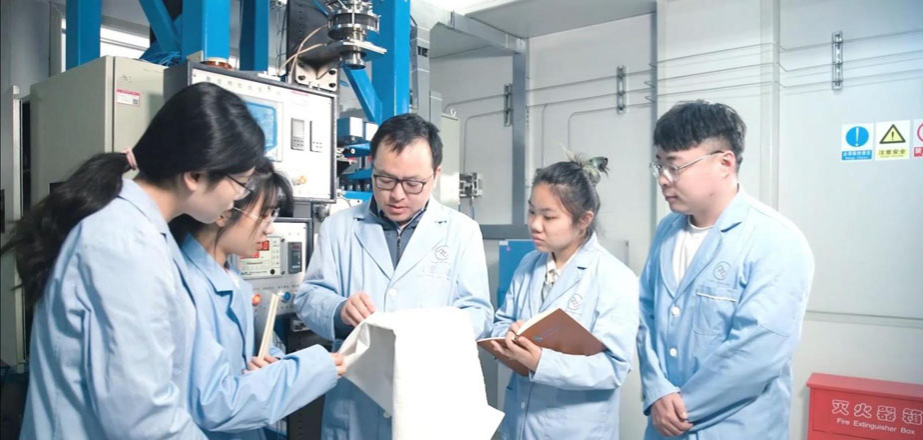
Prof. Tao (third from left) and his group members in intelligent fiber drawing tower laboratory

**Figure:**
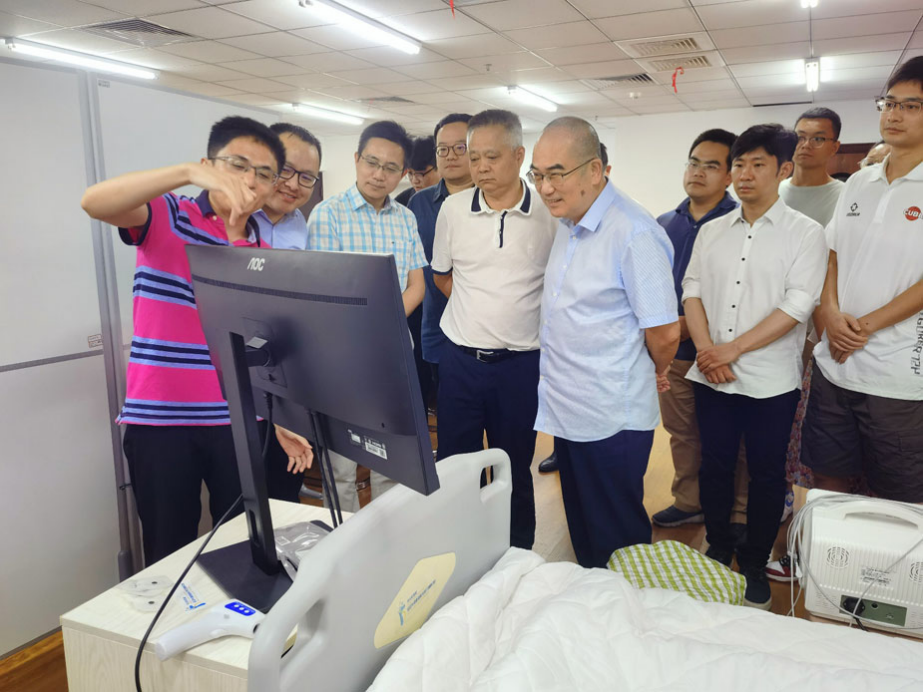
Prof. Tao (second from left) demonstrated the intelligent bed with a group of visitors led by Dr. Dingyu Zhang (sixth from left)

**2. In 2021, you and Associate Professor Yaoguang Ma of Zhejiang University collaborated for an article in** ***Science***^1^ **titled ‘Hierarchical-morphology metafabric for scalable passive daytime radiative cooling’. This work introduced an optical metafabric that achieved 92.4% solar radiation reflectivity and 94.5% mid-infrared emissivity and can cool the human skin by approximately 5 °C and the interior of a car by nearly 30 °C. What breakthroughs have been made in this study? What do you think are the development trends, application prospects, and future challenges of metafabrics? When do you think it will be available to ordinary people?**

Prof. Tao: Our metafabric technology is a universal outdoor cooling technique^2^. We developed a high-efficiency passive cooling metafabric through an industrial approach for mass production, which is promising for bringing cooling fabrics from the laboratory to daily life.

Since our article was published in *Science*, our group has received significant attention from academia. Scientists around the world have published several articles based on our metafabric work. In particular, an article written by Prof. Shuang Zhang in *Light: Science & Applications*^3^ stated that, ‘With its superior performance in radiative cooling performance, breathability, and wearing comfort, a wide range of applications can be envisioned for the designed metafabrics, including clothing, tents, and car covers’. Our work cannot be performed without significant support from the industry. Currently, we are promoting a combination of metafabric technology with the well-established manufacturing industry. Moreover, we have fabricated metafabrics based on modified polyester fibres, hoping to provide an innovative technology to improve the industrial chain. In addition, our metafabric technology is zero-energy and sustainable, which is in line with national strategies. Our future work will focus on achieving an even better integration with the manufacturing industry.

Many enterprises have communicated with us, hoping to adopt metafabric technologies. Metafabric applies not only to intelligent clothing but also to objects used outdoors, such as tents, and in construction activities and cold-chain logistics. For consumers, metafabric achieves zero-energy cooling while feeling almost as comfortable as everyday clothing. Our team continues to push this work forward and hopefully, metafabrics will be incorporated into our daily lives in the near future.

**Figure:**
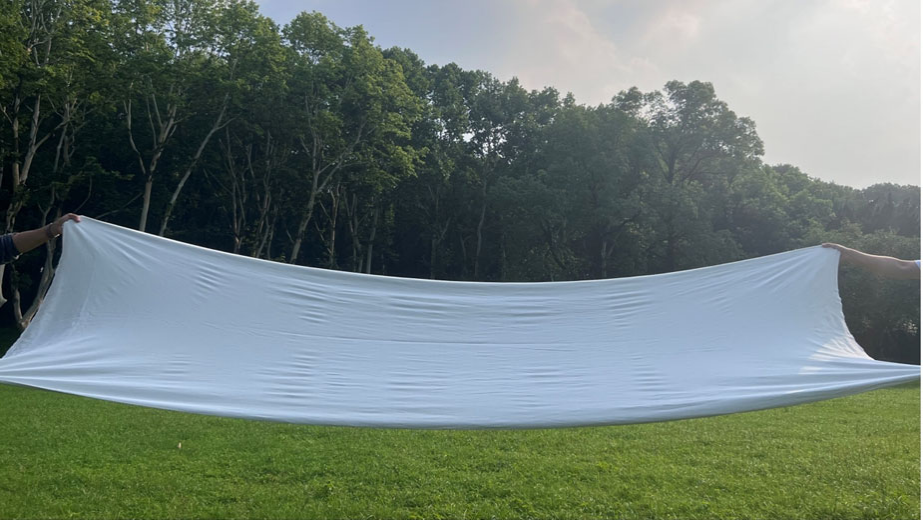
Massive-fabricated passive cooling metafabric based on modified polyester fibers (the latest result)

**3. The passive warm snow-face mask developed by your team was a hit among cross-country skiers at the Olympic Winter Games Beijing 2022. This technology not only won recognition from peers but also a letter of appreciation from the General Administration of Sport of China. What do you think of the industry–university–research cooperation? Could you talk about your experiences and feelings during the R&D process?**

Prof. Tao: Being involved in the ‘Science and Technology for the Winter Olympics’ project has inspired us significantly. Even though this project was small, we realised that research institutions and companies could collaborate more closely to complete the same mission. Interdisciplinary collaboration provides new strengths.

During the R&D process, there was no relevant domestic or international technology that could be used as reference. However, with the support of the General Administration of Sports of China, we successfully developed a passive warm snow face mask. We cooperated with several manufacturing companies to fabricate several batches of face shields for the national cross-country skiing team. We received expressions of gratitude from high-level national team experts and a Letter of Appreciation from the General Administration of Sports of China. This letter makes us feel that our work is valuable.

**Figure:**
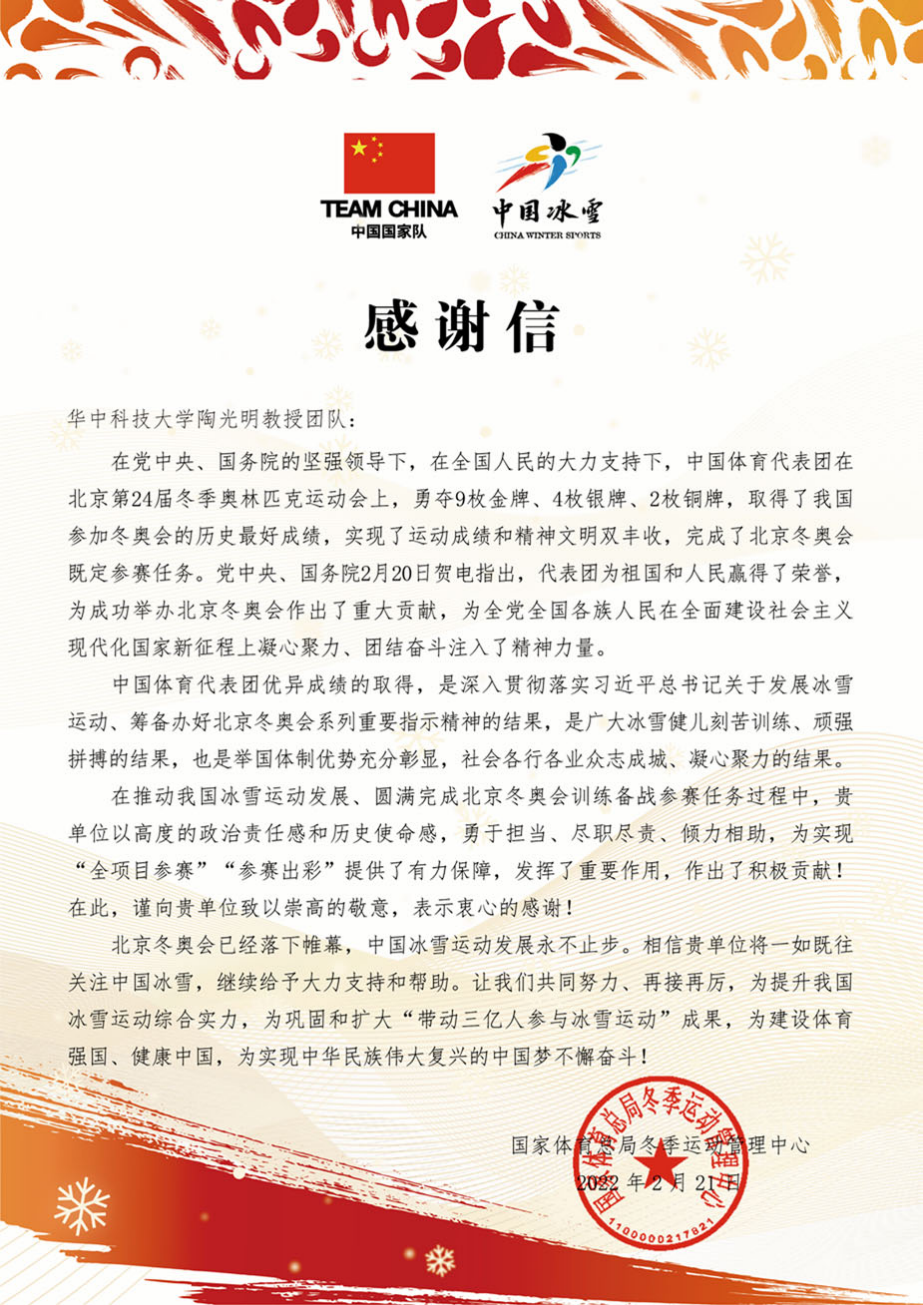
A Letter of Appreciation from the Winter Sports Management Center of the General Administration of Sports of China

**4. How do you view the relationship between research and commercialisation?**

Prof. Tao: I think this relationship is complementary. Only successful research can be commercialised, and commercialisation promotes better scientific and technological achievements, thus enhancing the core competitiveness of future industries.

**5. You studied at Shandong University and Fudan University before pursuing a PhD at the University of Central Florida. Why did you choose to return to China instead of staying in the United States? Did your experience in the United States affect your research direction after returning to China? In your opinion, what are the similarities and differences between China and the United States in education and research?**

Prof. Tao: I decided to return to China for two reasons—first, my family, and second, I believe my research should be better integrated with the manufacturing industry. As a young scientist, I hope to contribute to the upgradation of China’s manufacturing industry. My current research direction is also based on research conducted during my PhD. The United States is undoubtedly ahead in terms of education and research, but China is gradually improving and catching up.

**6. Once back in China, you have been working on several research projects as the principal investigator. How do you guide your research group? What capabilities do you focus on in student training?**

Prof. Tao: A scientific research project aims at solving realistic problems. My students have different educational backgrounds, and I must supervise them to conduct interdisciplinary research effectively. I frequently communicate with my students face-to-face to help them understand their perspectives on the research. Our research contributes to the development of society, as well as to the continuing cycle of science and technology.

Students have their own personalities, but at every stage of life, they need to do what they should do. I think the most important capabilities in the student stage include being dependable, hard-working, and faithful to what the student is working on. Furthermore, students should have the ambition, courage, and ability to challenge themselves. I hope that all my students can contribute as much as possible to society.

**Figure:**
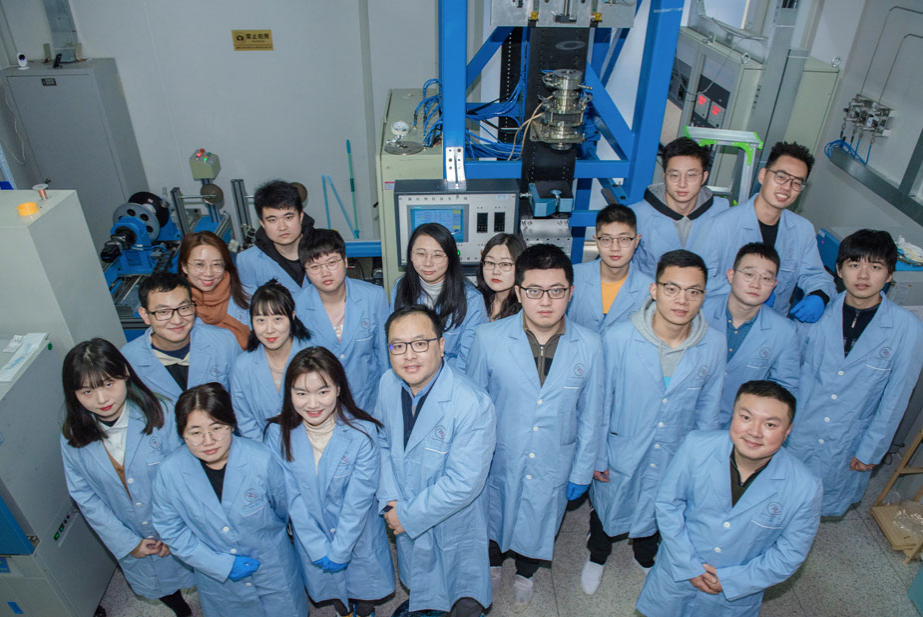
Partial members of Center for Fiber Optoelectronics Team led by Prof. Tao

**7. Having been engaged in the research on intelligent fibres and fabrics for many years, what is your evaluation of China’s development in this field? What areas do you think need to be strengthened?**

Prof. Tao: Chinese research groups have unearthed many groundbreaking findings in the field of intelligent fibres and fabrics, ushering in a trend of rapid progress in fundamental science. Nevertheless, the new challenge is how to make breakthroughs in fundamental science that can serve the manufacturing industry, and finally go on the shelves to serve people’s lives. Many practical engineering issues cannot be encountered or overcome in laboratory environment. This requires us to step out of the comfortable zone of the laboratory and bravely address the challenges encountered in the industry.

**8. What do you think is the significance of multidisciplinary integration in science and society? What difficulties and challenges did you encounter when switching between disciplines?**

Prof. Tao: In my research process, I gradually realised that a single discipline has inherent limits regarding practical problems. The most effective way to address these issues and challenges is through multidisciplinary integration and research collaboration. Therefore, we have established the Sports and Health Initiative at the Optical Valley Laboratory. The laboratory gathers researchers from multiple disciplines, such as optical engineering, materials science, computer science, medicine, mechanical engineering, and textile engineering, to promote interdisciplinary research.

It is difficult to break down the conceptual barriers between researchers with different educational backgrounds. Initially, young students cannot fully understand the cooperative relationships among multiple disciplines, but we are dedicated to helping them understand the idea of multidisciplinary integration, guiding them toward meaningful cooperation, and fostering systematic scientific thinking.

**9. What has been the most impressive or challenging aspect of your research career? How did you cope with and overcome it?**

Prof. Tao: Problems and challenges continue to appear in research. I think the most challenging moment was the early days of my return to China. At that time, the research platform was incomplete and many unexpected problems emerged. The key to overcoming these challenges was to have firm determination to help society. We should take advantage of our strengths and have the patience and optimism required to solve critical problems that hinder the development of society. Meanwhile, as researchers, it is important to think continuously about the frontier and try to find solutions.

**10. Has anyone had a major influence on you in your career? In what way?**

Prof. Tao: Howard Gardner, a professor of cognition and education at the Harvard Graduate School of Education, greatly influenced me. His theory of multiple intelligences defines intelligence as ‘the ability to solve problems, or to create products, that are valued within one or more cultural settings’. This is also my research concept. Our research focuses on solving a real problem in society or creating a valuable product for a specific group of people.

**11. What are your hobbies after work? How do you balance career and family?**

Prof. Tao: In my spare time, I explore and learn new things in interdisciplinary fields. I try to spend as much time as possible with my family, watching TV, eating delicious food, travelling, and so on. The company of my family provides me with spiritual comfort. My children are growing up, and I do not want to miss the important moments in their growth. Because of the nature of my research work, I do not have much time to stay with my family, but I am doing my best to spend my spare time with them.

**12. Based on your personal experiences, what do you think is a good researcher? What qualities do you think are essential?**

Prof. Tao: As a young researcher, I can share my limited experience with students; we should enjoy the research process, which is the key to becoming an excellent researcher. Meanwhile, success is only a matter of time if one chooses the right research direction, grasps the key questions, and maintains patience and diligence.

In my view, a researcher must have a strong faith, responsible attitude, and excellent teamwork skills. These qualities can help maintain focus, vitality, and innovative thinking in the research process.

**13. What advice or suggestions would you give our young readers on life and career?**

Prof. Tao: As a young scientist, I have been able to share some lessons in my research with students. First, to endure loneliness; self-discipline helps us achieve our purposes. Second, to bravely resolve academic challenges and pursue difficult goals. Finally, to maintain faith and persevere.

**Light special correspondent**

*WANG Hui is the Deputy Director of Department of International Cooperation in the Changchun Institute of Optics, Fine Mechanics and Physics (CIOMP), Chinese Academy of Sciences (CAS). She currently works on international communication and cooperation for the CIOMP and was a founding member of the journal Light: Science & Applications, which is a joint publication of Nature Publishing Group and CIOMP. She has published several articles in Acta Editologica, International Talent, Light: Science & Applications, etc., and was invited to contribute an article to SPIE Women in Optics in 2015. She is the initiator of the Rose in Science event and the co-sponsor and moderator of the iCANX Story. She has interviewed Donna Strickland, Nobel Laureate in Physics; Jean-Marie Lehn, Nobel Laureate in Chemistry; Johanna Stachel, the first female president of the German Physical Society; Chennupati Jagadish, president of the Australian Academy of Sciences; Carmen Menoni, president of the IEEE Photonics Society; Lin Li, Academician of the Royal Academy of Engineering; Zhonglin Wang, the first Chinese to receive the Eni Prize, etc.*

**Figure:**
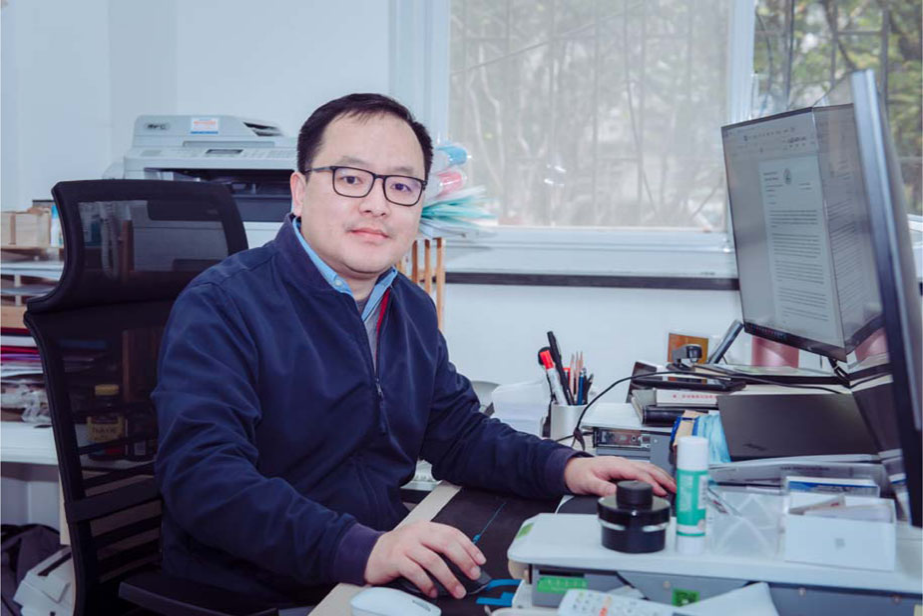
Prof. Guangming Tao devoted himself to the interdisciplinary research on fiber optoelectronics, and kept exploring thermal management fabrics and intelligent fabric space for sports and health.
